# Co‐Doped (Na^+^, Mg^2+^, Zn^2+^, Fe^3+^)‐Carbonated Hydroxyapatites and Composites with M^II^Fe_2_O_4_: Synthesis, Mechanic, Magnetic, and Antibacterial Properties for Medical Application

**DOI:** 10.1002/open.70232

**Published:** 2026-05-19

**Authors:** Yeva Komashchenko, Iryna Grynyuk, Dmytro Prokhorenko, Serhii Nedilko, Alla Kuryliuk, Roman Ostapenko, Olha Vasyliuk, Oksana Livitska, Nataliia Strutynska

**Affiliations:** ^1^ Chemistry Department Taras Shevchenko National University of Kyiv Kyiv Ukraine; ^2^ Department of Bioenergy, Bioinformatics and Environmental Biotechnology Igor Sikorsky Kyiv Polytechnic Institute Kyiv Ukraine; ^3^ Physics Department Taras Shevchenko National University of Kyiv Kyiv Ukraine; ^4^ Zabolotny Institute of Microbiology and Virology National Academy of Science of Ukraine Kyiv Ukraine; ^5^ Enamine Ltd Kyiv Ukraine

**Keywords:** biotechnology, composite, cytotoxicity, ferrite, hydroxyapatite

## Abstract

Co‐doped (Na^+^, Mg^2+^, Zn^2+^, Fe^3+^, CO_3_^2‐^)‐calcium phosphates and their composites with 10 or 25 wt% of (Mg/Zn)Fe_2_O_4_ were synthesized via a one‐step method from aqueous solutions and heated to 600°C. X‐ray powder diffraction (XRD) analysis confirmed the formation of single‐phase modified hydroxyapatites and biphasic calcium phosphate composites with a ferrite phase. The composite based on modified biphasic calcium phosphate with 10 wt% of ZnFe_2_O_4_ exhibited a higher hardness (HV = 3.28 GPa) than the corresponding co‐doped (Na^+^, Mg^2+^, Zn^2+^, Fe^3+^)‐hydroxyapatite (HV = 2.49 GPa) and a composite of modified biphasic calcium phosphate with 25 wt% ZnFe_2_O_4_ (HV = 2.91 GPa). The magnetic properties, studied by the Faraday method, showed that the composite with 25 wt% MgFe_2_O_4_ possessed a significantly higher magnetic susceptibility (345.9) than its ZnFe_2_O_4_ counterpart (77.8). Furthermore, an increase in the *β*‐Ca_3_(PO_4_)_2_‐based phase in the 25 wt% M^II^Fe_2_O_4_ composites was found to enhance the material's activity toward partial dissolution, a key factor in bone engineering. The antibacterial evaluation against *Staphylococcus aureus* and *Pseudomonas aeruginosa* demonstrated the functional potential of co‐doped (Na^+^, Mg^2+^, Zn^2+^, Fe^3+^, CO_3_^2‐^)‐hydroxyapatites and composites based on modified biphasic calcium phosphates with 25 wt% M^II^Fe_2_O_4_. These results contribute to the development of safe materials with tailored magnetic, mechanical, and biomedical properties.

## Graphical abstract

Multi‐doped (Na^+^, Mg^2+^, Zn^2+^, CO_3_^2‐^) hydroxyapatite/ferrite composites were synthesized via a one‐stage method. These “smart” materials exhibit high microhardness (3.28 GPa), potent antimicrobial activity, and membrane‐stabilizing effects, reducing erythrocyte hemolysis by up to 25%. Their tunable functional properties make them promising, infection‐resistant candidates for advanced bone tissue engineering and targeted therapy.

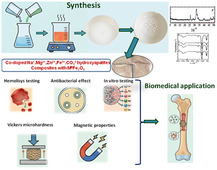

## 1 Introduction

Today magnetic nanoparticles, such as ferrite M^II^Fe_2_O_4_ are widely investigated for biomedical applications, including magnetic drug delivery [1, 2, 3, 4, 5], noninvasive magnetic resonance imaging (MRI) [6, 7], and magnetic hyperthermia [8, 9]. The influence of the size and chemical composition of nanostructured ferrites on their physicochemical properties is a critical factor in material design for specific applications. At the same time, the magnetic ferrites can exhibit cytotoxicity due to ion release, while coating them with a biocompatible shell reduces toxicity [10]. Thus, the formation of magnetic core–shell structures is an effective strategy for improving performance. Core–shell combinations, featuring a magnetic core and a compatible layer (polymers, silica, or others), are extensively proposed for designing “smart” and safe materials [10, 11, 12, 13, 14]. These systems can simultaneously generate heat and trigger drug release [13, 14]. For example, cytocompatibility of chitosan/Fe_3_O_4_ nanopowders was significantly enhanced by the coating, rendering them nontoxic and promising for drug carrying and delivery [10].

At the same time, the combination of ferrite with hydroxyapatite (Ca_10_(PO_4_)_6_(OH)_2_, HAP) represents a frontier in regenerative medicine. Hydroxyapatite mimics the mineral component of human bone, providing a scaffold that cells recognize. The ferrite component allows the scaffold to be stimulated by external magnetic fields, which has been shown to accelerate bone tissue regeneration and control cell growth orientation [15, 16, 17, 18, 19, 20]. A number of researchers have already demonstrated the external biophysical stimulations (ultrasound, magnetic pulse), which may act as an important factor for new bone cell regeneration [21, 22].

Thus, it was demonstrated that the potential of magnetic calcium phosphate combined with superparamagnetic iron oxide nanoparticles as bioactive magnetic scaffolds in bone tissue engineering. In the presence of a magnetic field, this material exhibited a synergistic effect that enhanced the osteogenic differentiation [23]. The hydroxyapatite functionalized *γ*‐Fe_2_O_3_ nanoparticles exhibited excellent biocompatibility with no observable toxicity and thus could potentially be used in magnetic hyperthermia therapy for cancer and bone regeneration as well as drug release applications [16, 24]. The hydroxyapatite coated iron oxide demonstrated a remarkably high efficiency in eliminating nearly all the experimental MG‐63 osteosarcoma cells [25]. These reported results indicate a new direction for biomaterial applications in anticancer therapies based on magnetic hyperthermia.

The development of biocompatible and biodegradable ionic substituted hydroxyapatite endowed with superparamagnetic and hyperthermia features via doping HAP with Fe^2+^/Fe^3+^ ions is also the perspective direction of materials design [26, 27, 28, 29]. Simultaneously, in vitro studies have demonstrated that Fe‐containing hydroxyapatite positively influences osteoblast‐like cells and mesenchymal stemcells viability [27, 28, 29] and is an effective carrier of the drug doxorubicin in the presence and absence of an applied low‐frequency electromagnetic field [26]. It was also shown that iron‐doped HAP samples had a higher solubility and dissolution rate than pure samples, which indicated that iron increased the bioactivity of HAP in vitro [30]. Today such magnetic hydroxyapatite nanoparticles can be obtained using different routes: chemical precipitation, sol–gel, mechanochemical, hydrothermal, and emulsion synthesis, template, microwave‐assisted, and biomimetic methods [31]. Each of them results in the particles formation with different sizes, shapes, crystallinity, magnetic behavior, solubility, bioactivity, etc. A combination of two of the listed methods (synergistic approach) allows to improve the desired properties of the future material.

Furthermore, the introduction of foreign cations such as Na^+^, Mg^2+^, Zn^2+^, Cu^2+^, as well as anion CO_3_^2−^ in the hydroxyapatite structure as a partial substitution of Ca^2+^ and PO_4_^3−^ and OH^‐^ allows for precise control over the stability and surface properties of the material [22]. Such doped nanoparticles are prospective materials with enhanced biological capabilities [32, 33, 34].

Recent studies have focused on improving the mechanical and biological performance of HAP‐based materials by creating composites with other oxides, including of rare‐earth oxides and carbon‐based nanomaterials. For instance, micro‐Vickers hardness tests for **Ca**_9.625_Na_0.25_Mg_0.25_ **(**PO_**4**_ **)**_**5.5**_ **(**BO_**3**_ **)**_**0.5**_ **(ВО**_**2**_ **)**_**2**_ and its composite with 25 wt% of ZrO_2_ showed an approximate two‐fold increase in hardness [35]. In a composite based on hydroxyapatite doped with Na^+^ (0.6 wt%), Mg^2+^ (0.6 wt%), Zn^2+^ (1.7 wt%), CO_3_^2‐^ (3 wt%), increasing the ZrO_2_ content from 10 to 25 wt% led to a 1.4‐fold increase in hardness [36]. For composites based on multidoped (Na^+^, Mg^2+^, Zn^2+^, BO_3_^3‐^/BO_2_^‐^) calcium phosphate, it was found that the addition of 10 wt% of ZrO_2_ caused an insignificant increase in hardness (HV) from 2.07 ± 0.10 to 2.13 ± 0.10 GPa, while 25 wt% ZrO_2_ raised the microhardness to 2.45 ± 0.11 [37]. At the same time, the addition of Gd_2_O_3_, Ho_2_O_3_, or Y_2_O_3_ in combination with graphene oxide has been shown to optimize surface topography and mechanical strength [38, 39, 40]. Similarly, ternary systems involving HAP, Cu_2_O, and graphene oxide significantly enhance antibacterial properties, making these composites ideal for biomedical applications [41].

The aim of this article was to synthesize multidoped (Na^+^, Mg^2+^, Zn^2+^, Fe^3+^, CO_3_^2‐^) calcium phosphates and their composites with variable amounts (10 and 25 wt%) of M^II^Fe_2_O_4_ (M^II^ – Mg^2+^, Zn^2+^) using a one‐step aqueous solution method. Furthermore, the study aimed to investigate the mechanical hardness, magnetic susceptibility, and antibacterial activity against *S. aureus* and *P. aeruginosa* of obtained multidoped hydroxyapatites and their composites with 10 or 25 wt% of M^II^Fe_2_O_4_.

## 2 Materials and Methods

### 2.1 Synthesis of Co‐doped Calcium Phosphates and Composites

Co‐doped (Na^+^, Mg^2+^, Zn^2+^) or Fe^3+^ cations‐carbonated calcium phosphates and their composites with 10 or 25 wt% of M^II^Fe_2_O_4_ (M^II^ – Mg^2+^, Zn^2+^) were prepared from aqueous solutions at different molar ratios of components in initial aqueous solutions and further heated to 600°C (Table 1).

**TABLE 1:** The molar ratios of components at fixed ratio PO_4_^3‐^: CO_3_^2‐^ = 2 : 2 in initial mixtures and codes of samples.

Codes	Molar ratios in initial mixtures					Sample composition	
	Ca^2+^	Fe^3+^	Zn^2+^	Mg^2+^	Na^+^	Substitution, mol%	Ferrite, wt%
2.5FeHAP	37.5	1				2.5 Fe	—
5FeHAP	36.0	2	—	—	—	5Fe	—
2.5(ZnMgNa)HAP	36.5	—	1	1	1	2.5(ZnMgNa)	
5Zn2.5(MgNa)HAP	35.5	—	2	1	1	5Zn2.5(MgNa)	
2.5(FeZnMgNa)HAP	35.0	1	1	1	1	2.5(FeZnMgNa)	
2.5(MgNa)HAP‐10ZnFe	37.5	4	2	1	1	2.5(MgNa)	10ZnFe_2_O_4_
2.5(MgNa)BCP‐25ZnFe	37.5	10	5	1	1	2.5(MgNa)	25ZnFe_2_O_4_
2.5(ZnNa)BCP‐10MgFe	37.5	4	1	2	1	2.5(ZnNa)	10MgFe_2_O_4_
2.5(ZnNa)BCP‐25MgFe	37.5	12	1	6	1	2.5(ZnNa)	25MgFe_2_O_4_

*Note:* C(Ca^2+^) = 0.1 M.

The following substances Ca(NO_3_)_2_ · 4H_2_O, Mg(NO_3_)_2_·6H_2_O, Zn(NO_3_)_2_ · 6H_2_O, NaNO_3_, Fe(NO_3_)_3_·9H_2_O, (NH_4_)_2_HPO_4_, NH_4_HCO_3_ (an analytical grade), and NH_3_·H_2_O (25%) were used as starting components. The calculated amount of initial components were dissolved in 25 mL of deionized water. After mixing the solution that contained metals nitrate with phosphate‐carbonate containing solution and adding ammonia (3 mL), the water was evaporated and solids were heated to 600°C for 2 h. The obtained powders were grounded and investigated.

### 2.2 Methods of Characterization

X‐ray powder diffraction (XRD) method (a diffractometer Shimadzu XRD‐6000, CuKα radiation, *λ* = 1.54178 Å) was used for phase analysis, calculation of lattice parameters, and crystallites size of prepared samples. The patterns were collected at 2*θ* range from 5° to 60° with the step of 0.02°. Stoichiometric hydroxyapatite (ICSD #00‐074‐0565) was used for identification of apatite‐type phase. The crystallites size was determined from the broadenings of corresponding X‐ray spectral peaks by using Scherrer's formula:

D=0.94 λ/(βcosθ)

where *λ* is the X‐ray wavelength, *θ* is the Bragg diffraction angle, and *β* is the FWHM of the XRD peak appearing at the diffraction angle *θ* of prominent peak at (101).

Fourier‐transform infrared spectroscopy (FTIR, PerkinElmer Spectrum BX spectrometer) was used for confirmation of the presence of different anions in the prepared samples. The wavenumber range was 400–4000 cm^−1^.

### 2.3 In Vitro Activity

The influence of synthesized modified calcium phosphates and composites with different chemical and phase compositions on pH of model solution was determined at a temperature of 37°C and pH = 7.45. The determined sample (0.2 g) was immersed in 15 mL of solution at 37°С for 3 days. The value of pH was measured after 1 h, 24, 48, and 72 h using a рН meter (OHAUS Started 2100).

### 2.4 Antibacterial Properties

The effect of synthesized powders 2.5FeHAP, 5FeHAP, 2.5(ZnMgNa)HAP, 5Zn2.5(MgNa)HAP, 2.5(FeZnMgNa)HAP, 2.5(MgNa)BCP‐25ZnFe, and 2.5(ZnNa)BCP‐25MgFe against opportunistic microorganisms *Staphylococcus aureus* (ATCC 25 923) and *Pseudomonas aeruginosa* (АТСС 9027) was investigated as described in [42]. Determined samples before investigation were sterilized by autoclaving at 0.75 atm and temperature 112°C for 30 min. Strains of microorganisms were cultured two times on tryptone soya broth at 37°C during 24 h. After that, the different amounts (5, 10, and 20 mg/mL) of powder were added in sterile bottles with nutrient medium and 2% of the overnight culture *S. aureus* or *P. aeruginosa* (10^5^ CFU/mL). As a control, the growth of culture without powder was used. The bottles were incubated at 37°C ± 1°C for 24 h, and then suspensions of 24‐hour bacterial culture with some samples were incubated on petri dishes containing tryptone soya agar. On the next day, the amount of bacterial colonies of tested strains on the surface of the agar medium was calculated.

Data processing was performed using the program ≪STATISTICA 7.0≫ (StatSoft Inc., Tulsa, OK, USA). The posthoc‐test using the criterion of LSD was used for assessing the reliability of quantitative indicators of differences in different strains. Statistical significance was defined as at *p* ≤ 0.05. Antibacterial data were presented as the mean (M) ± standard deviation (SD). Each experiment was repeated four times.

### 2.5 Hemolisys Testing

Erythrocytes were obtained from heparinized blood of the ram. The animals were kept under standard conditions in the vivarium of the ESC “Institute of Biology and Medicine”, Taras Shevchenko National University of Kyiv. Animals had free access to food and water. The Committee on Bioethics of Scientific Research of Taras Shevchenko National University of Kyiv (Kyiv, Ukraine) approved the method of hemolysis of animal erythrocytes (ram) to study the cytotoxicity of the tested samples (protocol №8 dated December 26, 2024), adhering to the criteria of international standards (European Convention dated September 22–24, 2004 “On the protection of animals used for experimental and other scientific purposes", the Directive of the European Parliament and the Council of the European Union dated September 22, 2010 “On the protection of animals used for scientific purposes") and current legislation (the Law of Ukraine “On the Protection of Animals from Cruelty” dated February 21, 2006 №3447‐IV (dated February 13, 2020)).

For hemolysis estimation, erythrocytes were diluted in 0.85% NaCl solution to 0.700 o.u. at 630 nm on a spectrophotometer (ULAB, China).

Erythrocytes were incubated in 0.85% NaCl solution without sample or with obtained samples in the amount of (1–5 mg/mL) during 30 min. Hemolysis of erythrocytes was caused by 0.001 N HCl. The kinetics of hemolysis were measured spectrophotometrically (*λ* = 630 nm) every 10 s during 2 min. The percentage of hemolyzed erythrocytes was calculated as presented in [43]. The obtained extinction data allowed us to construct kinetic hemolysis curves (erythrograms) and calculate the percentage level of cell lysis using the formula:

$$G=\frac{D_{i}-D_{i-1}}{D_{0}-D_{\infty }}\times 100\%$$

where *D* is the the optical density, *D*_0_ та *D*_∞_ are its initial and final values, *i* is the measurement serial number.

### 2.6 The Vickers Microhardness

The microhardness of the prepared powders was measured using a digital microhardness tester LHVS‐1000Z (Chongqing Leeb Instrument Co., Ltd., China) equipped with a standard diamond pyramid indenter. For each specimen, 50 indentations were made under a load of 0.02 kg applied for 10 s. After unloading, the diagonals of the impressions were measured, and the Vickers hardness (HV) was calculated according to the following expression:

$$\mathrm{HV}=\frac{1.854\times P}{d^{2}}$$

where *P* is the applied load in kilograms‐force and *d* is the average length of the diagonals of the indentation in micrometers.

### 2.7 Magnetic Properties

The magnetic characteristics of iron‐doped hydroxyapatites (2.5FeHAP and 2.5(FeZnMgNa)HAP) and composites based on biphasic calcium phosphates with 25 wt% of ZnFe_2_O_4_ (2.5(MgNa)BCP‐25ZnFe) or 25 wt% of MgFe_2_O_4_ (2.5(ZnNa)BCP‐25MgFe) were measured using Faraday's magnetic method under a magnetic field of 550 kA/m. This method is simple, universal, and based on the interaction between a substance and a nonuniform magnetic field [44]. In this technique, the sample is placed between electromagnet cores where a magnetic field is applied. The magnetometer is calibrated against a standard with known susceptibility. After measuring, the magnetic susceptibility (χ) was calculated using the following relation:

$$\chi =\chi_{\mathrm{ET}}\frac{m_{\mathrm{ET}}}{m}\frac{F_{z}}{F_{Z_{\mathrm{ET}}}}$$

χ, χ_**ET**_, is magnetic susceptibility, *m*, *m*_**ET**_ is mass, and *F* *z*, *Fz*_**ET**_ are forces which act from an inhomogeneous field on the sample and the standard, respectively. The details of the method can be seen in the article [44].

## 3 Results and Discussion

### 3.1 Sample Characterization

Nine samples with different phase and chemical compositions were synthesized from aqueous solution at different molar ratios of components in initial mixtures (Table 1) and subsequently heated to 600°C. These ratios were designed to achieve different types of partial substitution of calcium atoms by foreign cations in the cationic sublattice of the apatite‐type structure (Ca_10_(PO_4_)_6_(OH)_2_) as follows:

- $(10‐3x){\mathrm{Ca}}^{2+}\to 2x{\mathrm{Fe}}^{3+}$ (Samples 2.5FeHAP and 5FeHAP);
- $(10-x-y-z/2)\;Ca^{2+}\to x{\mathrm{Zn}}^{2+}+yMg^{2+}+zNa^{+}$ (Samples 2.5(ZnMgNa)HAP and 5Zn2.5(MgNa)HAP);
- $(10‐x‐y‐z‐a){\mathrm{Ca}}^{2+}\to {\mathrm{aFe}}^{3+}+x{\mathrm{Zn}}^{2+}+y{\mathrm{Mg}}^{2+}+z{\mathrm{Na}}^{+}$ (Sample 2.5(FeZnMgNa)HAP)

and fixed partial substitution of phosphate by carbonate anions as well as the formation of composites based on modified carbonated calcium phosphates with 10 or 25 wt% of M^II^Fe_2_O_4_, Samples 2.5(MgNa)HAP‐10ZnFe and 2.5(ZnNa)BCP‐10MgFe or 2.5(MgNa)BCP‐25ZnFe and 2.5(ZnNa)BCP‐25MgFe), respectively.

**FIGURE 1:**
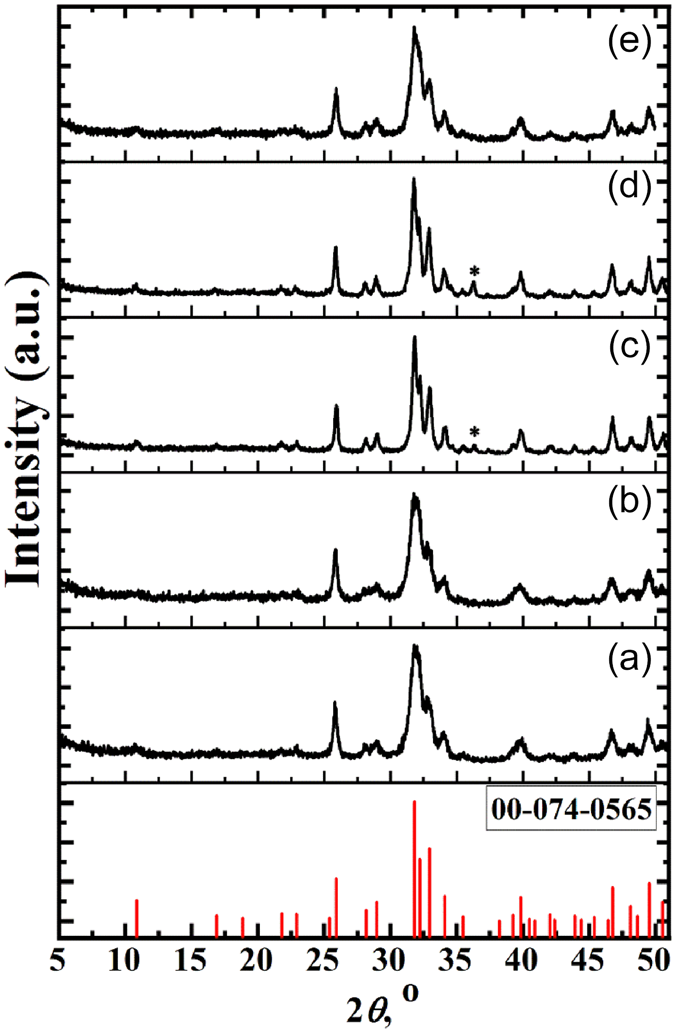
XRD patterns of synthesized modified hydroxyapatites: a) 2.5FeHAP, b) 5FeHAP, c) 2.5(ZnMgNa)HAP, d) 5Zn2.5(MgNa)HAP, and e) 2.5(FeZnMgNa)HAP heated at 600°С for 2 h and reference pattern for Ca_10_(PO_4_)_6_(OH)_2_(#00‐074−0565).

According to powder XRD, single apatite‐related phases were obtained only in the case of samples where calcium cations were partially substituted by other cations (Samples 2.5FeHAP, 5FeHAP, 2.5(FeZnMgNa)HAP) with the exception of Samples 2.5(ZnMgNa)HAP and 5Zn2.5(MgNa)HAP which contained an insignificant amount of ZnO impurities (Figure 1). The ZnO content increased with the concentration of Zn^2+^ in the initial solutions (Figure 1c,d, where ZnO is indicated by *).

**FIGURE 2:**
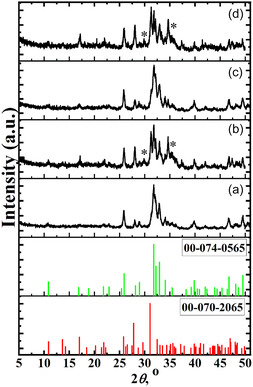
XRD patterns of prepared composites based on modified calcium phosphates with ferrites: a) 2.5(MgNa)HAP ‐10ZnFe, b) 2.5(MgNa)BCP‐25ZnFe, c) 2.5(ZnNa)BCP‐10MgFe, and d) 2.5(ZnNa)BCP‐25MgFe heated at 600°С for 2 h and reference patterns for Ca_10_(PO_4_)_6_(OH)_2_ (#00‐074‐0565) and *β*‐Ca_3_(PO_4_)_2_ (#00‐070−2065), *shows ferrite‐type phase.

In the “modified calcium phosphate/M^II^Fe_2_O_4_” systems, the formation of composites (modified hydroxyapatite/ferrite) was observed only for Sample 2.5(MgNa)HAP‐10ZnFe (Figure 2a). Conversely, the addition of Fe^3+^ and Mg^2+^ to the initial solution for the formation of composites with 10 or 25 wt% of M^II^Fe_2_O_4_ (Samples 2.5(ZnNa)BCP‐10MgFe, 2.5(MgNa)BCP‐25ZnFe, 2.5(ZnNa)BCP‐25MgFe) resulted in biphasic calcium phosphates (BCP ‐ mixture of phases based on Ca_10_(PO_4_)_6_(OH)_2_ and *β*‐Ca_3_(PO_4_)_2_) (Figure 2b–d). These data indicate that increasing the concentration of Fe^3+^ and M^2+^ in an initial solution triggers the formation of modified biphasic calcium phosphates. A similar effect of M^2+^ codoping leading to BCP formation has been previously reported [45, 46]. In this study the composites based on modified biphasic calcium phosphate with 10 or 25 wt% of M^II^Fe_2_O_4_ were obtained for the first time.

The calculated lattice parameters and crystallites size for the apatite‐type phases are summarized in Table 2. Comparison of lattice parameters of the prepared calcium phosphates with standard stoichiometric Ca_10_(PO_4_)_6_(OH)_2_ (*a* = 9.418 Å, *c* = 6.884 Å) [47] showed shifts in values, confirming successful substitution within the apatite structure.

**TABLE 2:** Calculated lattice parameters and crystallites size for synthesized modified hydroxyapatites (hexagonal system, space group*Р6*_3_ */m*).

Sample	Parameters		Crystallite size, nm
	*а*, Å	*c*, Å	
2.5FeHAP	9.446(6)	6.886(9)	25
5FeHAP	9.411(3)	6.884(9)	23
2.5(ZnMgNa)HAP	9.409(7)	6.864(1)	37
5Zn2.5(MgNa)HAP	9.424(5)	6.892(1)	35
2.5(FeZnMgNa)HAP	9.508(1)	6.883(6)	23

Analysis of the crystallites sizes revealed that the partial substitution of calcium by Fe^3+^ led to the formation of smaller particles (25 nm) compared to phosphates with the complex substitution by (Zn^2+^+ Mg^2+^+ Na^+^) ions (35–37 nm) (Table 2).

**FIGURE 3:**
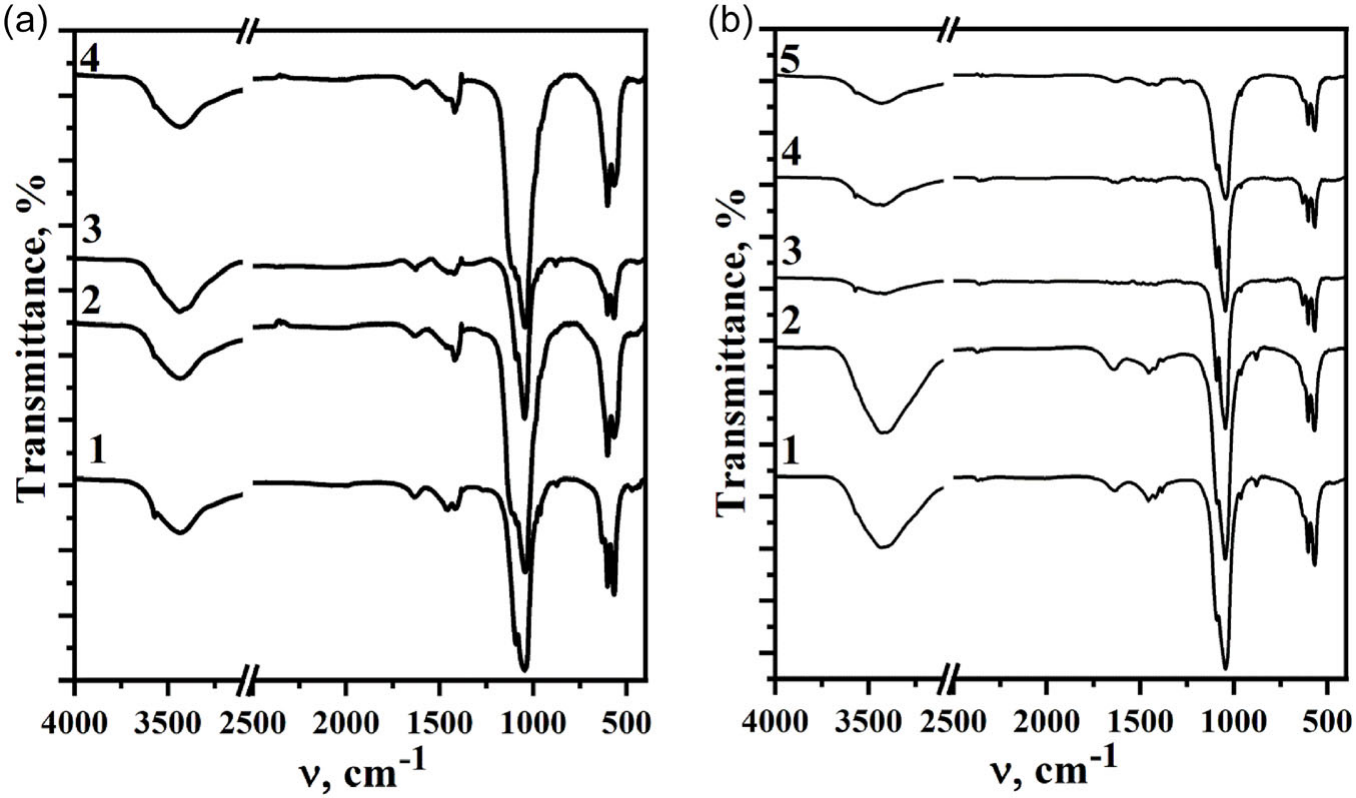
FTIR spectra of prepared samples (a) – 2.5FeHAP (curve 1), 5FeHAP (curve 2), 2.5(ZnMgNa)HAP (curve 3), 5Zn2.5(MgNa)HAP (curve 4) and (b) –2.5(MgNa)HAP‐10ZnFe (curve 1), 2.5(MgNa)BCP‐25ZnFe (curve 2), 2.5(ZnNa)BCP‐10MgFe (curve 3), 2.5(ZnNa)BCP‐25MgFe (curve 4), and 2.5(FeZnMgNa)HAP (curve 5) heated at 600°С for 2 h.

The FTIR spectra for all prepared samples exhibited similar vibration modes in terms of position and intensity (Figure 3). The main characteristic bands of phosphate group (PO_4_‐tetrahedra) were detected at 472, 567, 962, and 1060 cm^−1^. The typical hydroxyl (OH^−^) group bands were observed at 3650 and 632 cm^−1^ while broad bands in the regions 3300−3550 and 1610–1630 cm^−1^ correspond to the vibration modes of adsorbed water. The presence of typical CO_3_^2‐^ bands at 870–875, 1410−1430 and 1450–1470 cm^−1^ confirms the formation of B‐type carbonated hydroxyapatites, where carbonate groups partially substitute phosphate anions (Figure 3).

### 3.2 Vickers Microhardness Testing

**FIGURE 4:**
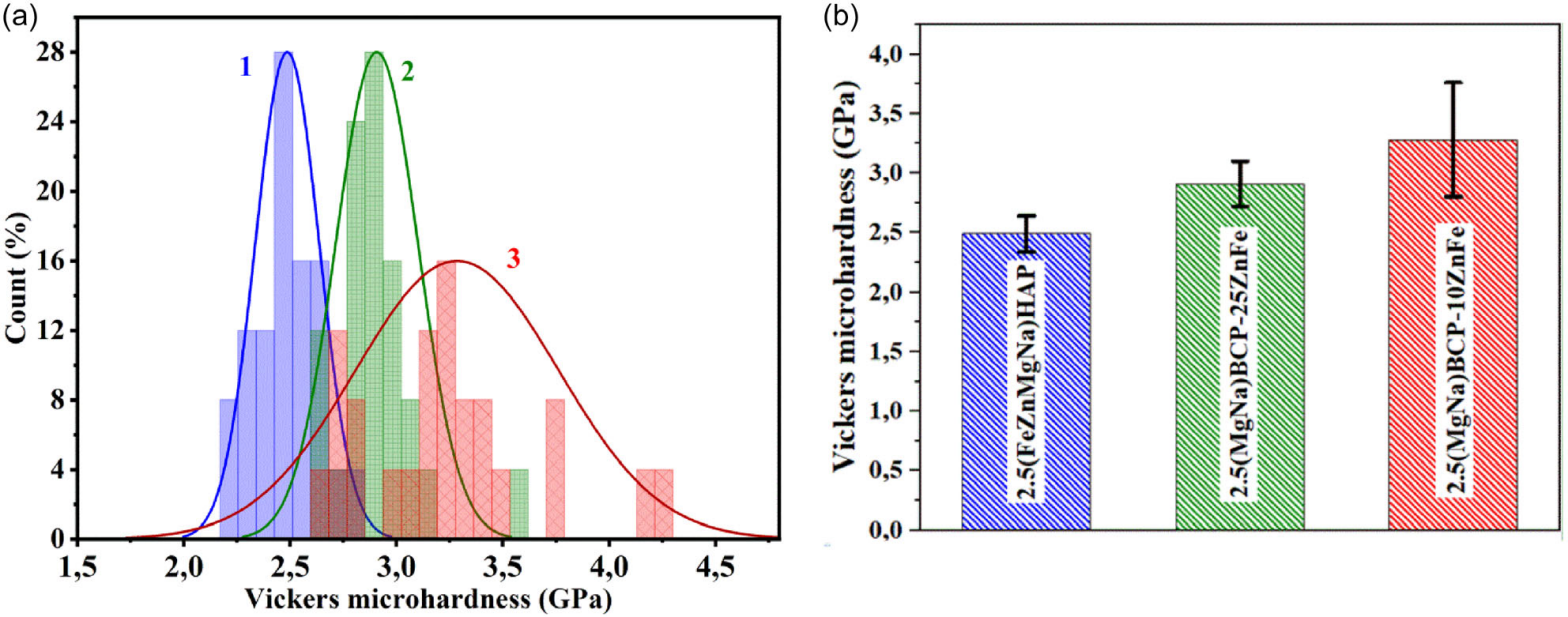
a) Histogram of the microhardness distribution and b) bar graph of hardness values for synthesized powders: 2.5(FeZnMgNa)HAP (curve 1), 2.5(MgNa)BCP‐25ZnFe (curve 2), and 2.5(MgNa)HAP‐10ZnFe (curve 3).

Figure 4 shows the histograms of the Vickers microhardness distribution for 2.5(FeZnMgNa)HAP (codoped (Na^+^, Mg^2+^, Zn^2+^, Fe^3+^)‐hydroxyapatite), 2.5(MgNa)HAP ‐10ZnFe (co‐doped (Na^+^, Mg^2+^)‐hydroxyapatite with 10 wt% ZnFe_2_O_4_), and 2.5(MgNa)BCP‐25ZnFe (co‐doped (Na^+^, Mg^2+^)‐biphasic calcium phosphate with 25 wt% ZnFe_2_O_4_). The lowest microhardness was observed for the 2.5(FeZnMgNa)HAP powder, with a most probable value of HV = 2.49 GPa. Sample 2.5(MgNa)BCP‐25ZnFe exhibits a slightly higher hardness of HV = 2.91 GPa, while for Sample 2.5(MgNa)HAP‐10ZnFe, which contains only 10 wt% of ZnFe_2_O_4_, this parameter reaches HV = 3.28 GPa. This result indicates that the presence of 10 wt% ferrite in composite with modified hydroxyapatite significantly increases its microhardness. It is also evident that the increase in hardness is accompanied by a broader distribution of values across the sample surfaces. In particular, the standard deviations are *σ*_HV_ = 0.15, 0.19, and 0.48 GPa for 2.5(FeZnMgNa)HAP, 2.5(MgNa)BCP‐25ZnFe, and 2.5(MgNa)BCP‐10ZnFe, respectively. This trend indicates an increase in the microstructural and/or chemical heterogeneity of the material, which is more pronounced in the HAP‐ferrite composite than in the HAP‐*β*‐Ca_3_(PO_4_)_2_‐ferrite (BCP‐ferrite) composite. The increase in microhardness for 2.5(FeZnMgNa)HAP (2.49 GPa) compared to previously reported multidoped (Na^+^, Mg^2+^, Zn^2+^, BO_3_^3‐^/BO_2_^‐^) hydroxyapatite (2.07 GPa) [37] highlights a critical point: the mechanical profile of nanohydroxyapatite is highly sensitive to its chemical composition. While the formation of composites is a traditional method for enhancing mechanical characteristics, these results demonstrate that simultaneous substitutions in both the cationic and anionic sublattices of calcium phosphate serve as a powerful alternative for tuning material performance. Consequently, the strategic “doping” of the hydroxyapatite lattice allows for the precise engineering of mechanical characteristics without the need for a secondary phase.

### 3.3 Magnetic Properties Analysis

The magnetic properties of the synthesized materials are intrinsically linked to the phase composition and the distribution of metal cations within the spinel lattice. The composite containing 25 wt% MgFe_2_O_4_ exhibited a significantly higher magnetic susceptibility (345.9) compared to the ZnFe_2_O_4_ equivalent (77.8). This disparity is attributed to the structural differences between these two ferrites: MgFe_2_O_4_ typically adopts an inverse spinel structure, which promotes strong superexchange interactions between Fe^3+^ ions in tetrahedral and octahedral sites, resulting in high ferrimagnetic induction. Conversely, ZnFe_2_O_4_ prefers a normal spinel configuration, where the absence of magnetic ions in tetrahedral sites weakens the magnetic ordering at room temperature. Observed values correspond to known data on the magnetic characteristics of these ferrites, both in their isolated states and in the composites [48, 49, 50, 51].

The absence of magnetic characteristics in the Fe‐modified hydroxyapatite further confirms that the Fe^3+^ ions are stabilized within the hexagonal HAP lattice as substitutional dopants. Under the specified synthesis conditions, no precipitation of magnetic iron oxide phases (like Fe_2_O_3_ or Fe_3_O_4_) occurred. Consequently, the proposed one‐stage technique provides a precise tool for tailoring the magnetic response of the composite by selecting the appropriate bivalent metal (Mg^2+^ vs. Zn^2+^) and controlling the degree of ferrite phase formation. This magnetic functionality is essential for potential applications in magnetic‐field‐assisted bone tissue regeneration.

### 3.4 In Vitro Testing

**FIGURE 5:**
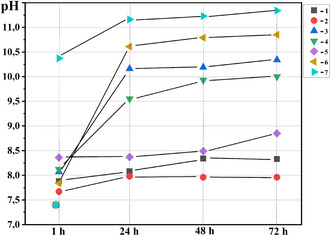
Change of pH value of model solution (at temperature 37°C and initial value of pH = 7.45) in the presence of obtained modified hydroxyapatites and composites based on modified calcium phosphate with M^II^Fe_2_O_4_ (10 or 25 wt%) during 72 h. 2.5FeHAP (curve 1), 5FeHAP (curve 2), 2.5(ZnMgNa)HAP (curve 3), 5Zn2.5(MgNa)HAP (curve 4), 2.5(FeZnMgNa)HAP (curve 5), 2.5(MgNa)HAP‐10ZnFe (curve 6), and 2.5(MgNa)BCP‐25ZnFe (curve 7).

Investigation of the prepared codoped hydroxyapatites and composites based on modified calcium phosphates with 10 or 25 wt% of M^II^Fe_2_O_4_ in a model solution (pH = 7.45 and temperature 37°C) showed a significant influence of their compositions on the character of pH value changes (Figure 5). The increase in pH values in the presence of all tested samples is caused by their partial dissolution.

The highest pH increase after 1 h was observed for sample 2.5(MgNa)BCP‐25ZnFe that contained a significant amount of the phase based on *β*‐Ca_3_(PO_4_)_2_, reaching pH 10.5; for the remaining samples, pH values ranged between 7.55 and 8.5 depending on their composition (Figure 5). During the subsequent 24 h, the nature of these changes differed significantly (Figure 5). The least influence with a pH increase from 7.45 to only 8.0 was found for Fe‐modified hydroxyapatites (2.5FeHAP and 5FeHAP) during the first 24 h. This remained almost unchanged for Fe‐rich samples (5FeHAP) over 72 h. Simultaneously, for the 2.5FeHAP sample, a pH increase to 8.3 occured at the 48‐hour mark of testing. In contrast, the multidoped (Na^+^, Mg^2+^, Zn^2+^, Fe^3+^, CO_3_^2‐^)‐hydroxyapatite (2.5(FeZnMgNa)HAP) showed a pH increase to 8.5 (at 48 h) and 8.8 (at 72 h) that indicates the role of complex trace elements (Na^+^, Mg^2+^, Zn^2+^) in regulating the activity of the synthetic material (Figure 5). This conclusion was additionally confirmed by the results for sample without Fe^3+^. Thus, in the case of codoped (Na^+^, Mg^2+^, Zn^2+^, CO_3_^2‐^)‐HAP, a pH increase to 10 after the first 24 h of treatment in solution was observed (Figure 5, curve 3 and curve 4). It should be noted that an increase of Zn^2+^ amount in an initial solution led to a decrease in sample activity. This fact may be caused by a reduced amount of Zn^2+^‐intercalation into the apatite structure, correlating with XRD data regarding the formation of ZnO impurity (Figure 5, curve 4). The most significant pH increase (above 10.5) after the first 24 h of soaking in model solution was found for composites based on modified hydroxyapatite with 10 wt% of ZnFe_2_O_4_ (Sample 2.5(MgNa)HAP ‐10ZnFe) and a composite based on modified biphasic calcium phosphate with 25 wt% of ZnFe_2_O_4_ (Sample 2.5(MgNa)BCP‐25ZnFe) (Figure 5, curves 6 and 7, respectively). This result indicates that the highest pH growth was caused by partial dissolution of composite with higher amount of bioactive phase based on *β*‐Ca_3_(PO_4_)_2_ in its composition. Thus, the obtained results show the possibility of local regulation of pH depending on the nature of the doping ions, while composites with modified biphasic calcium phosphate possess a high alkaline reserve. This property, combined with the controlled release of doping ions, is of particular importance for bone tissue engineering in the context of metabolic acidosis (pH 5.5–6.5). The growth of pH value during the partial dissolution of the bioactive phases serves to neutralize the acidic environment of the inflammatory focus. This buffering effect can prevent further degradation of the bone matrix and may alleviate localized pain. Simultaneously, the localized increase in pH to values near 10 creates a hostile environment for common bone pathogens. This effect is significantly amplified by the intrinsic antimicrobial properties of Zn^2+^ ions, which disrupt bacterial cell membranes and enzymatic functions, providing a dual‐action defense against postoperative infections. The inclusion of doping into the newly formed mineral layer can improve its properties and biological affinity compared to stoichiometric hydroxyapatite.

### 3.5 Antibacterial Effect

**FIGURE 6:**
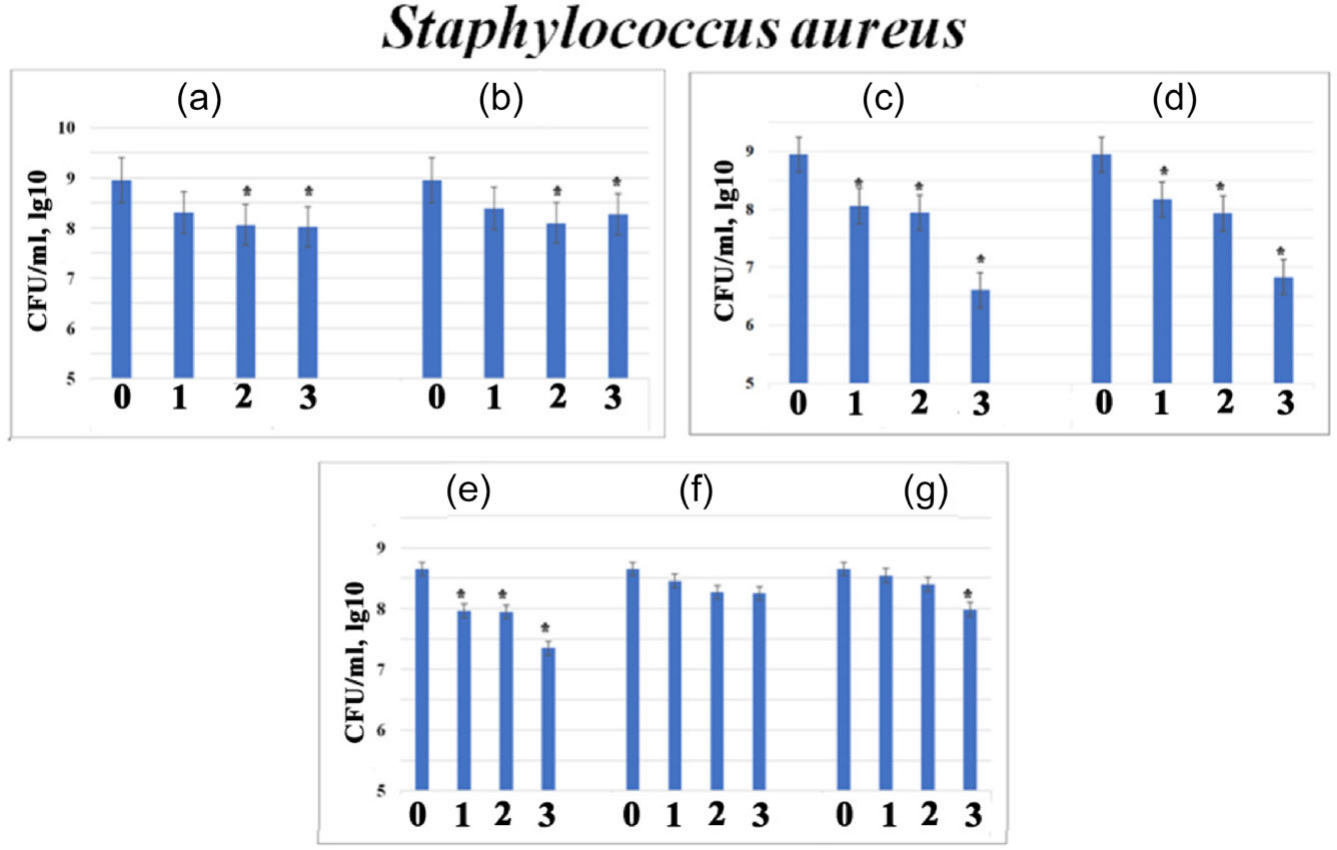
Antibacterial activity against *Staphylococcus aureus* ATCC for different amounts (5 mg/mL – (1), 10 mg/mL – (2) and 20 mg/mL – (3)) of prepared powders: 2.5FeHAP (a); 5FeHAP (b); 2.5(ZnMgNa)HAP (c); 5Zn2.5(MgNa)HAP (d); 2.5(FeZnMgNa)HAP (e); 2.5(MgNa)BCP‐25ZnFe (f); and 2.5(ZnNa)BCP‐25MgFe (g) (M ±m, *n* = 4, * – *p* < 0.05 compared to control).

**FIGURE 7:**
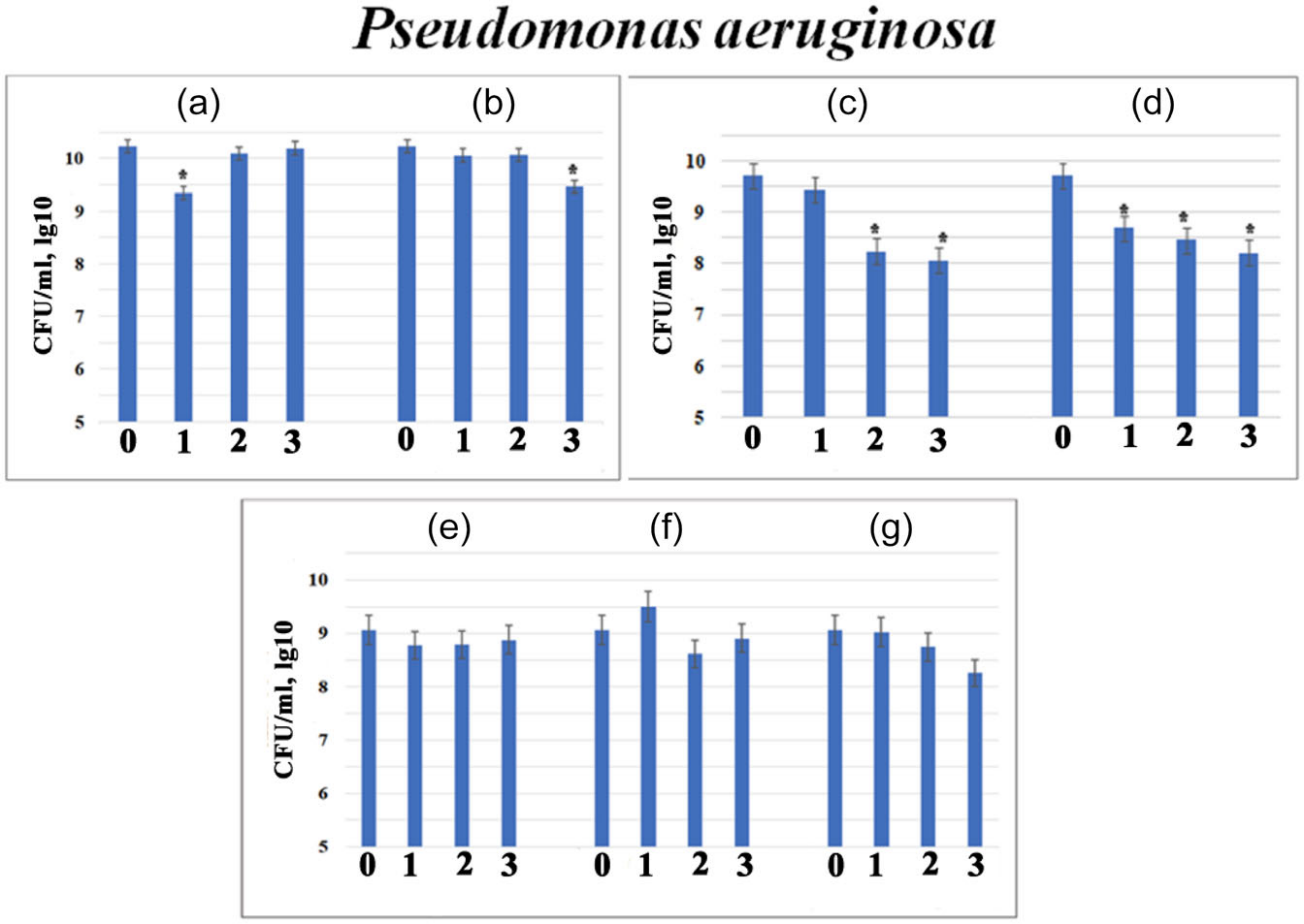
Antibacterial activity against *Pseudomonas aeruginosa* АТСС 9027 for different amounts (5 mg/mL – (1), 10 mg/mL – (2) and 20 mg/mL – (3)) of prepared powders: 2.5FeHAP (a); 5FeHAP (b); 2.5(ZnMgNa)HAP (c); 5Zn2.5(MgNa)HAP (d); 2.5(FeZnMgNa)HAP (e); 2.5(MgNa)BCP‐25ZnFe (f); and 2.5(ZnNa)BCP‐25MgFe (g) (M ±m, *n* = 4,* – *p* < 0.05 compared to control).

The antibacterial influence of modified calcium phosphates and composites against *Staphylococcus aureus* and *Pseudomonas aeruginosa* was investigated by adding variable amounts (5, 10, or 20 mg/mL) of the samples (Figures 6 and 7). The results revealed a clear correlation between the sample composition, the resulting alkaline shift in the medium, and the degree of growth inhibition.

#### 3.5.1 Activity Against Staphylococcus aureus

For Fe^3+^‐doped samples, an increase in iron content from 2.5 to 5.0 mol% had only a slight impact on *S. aureus* (Figure 6a,b). Specifically, the growth was inhibited by 4.6–4.0 times for 2.5FeHAP and 5.6–4.9 times for 5FeHAP. This relatively weak effect correlates with the previously observed insignificant pH shift for these samples, which remained near the physiological range.

In contrast, the highest antibacterial effect was recorded for co‐doped (Na^+^, Mg^2+^, Zn^2+^)‐hydroxyapatites. At a dose of 20 mg/mL, these samples induced a massive decrease in bacterial growth: 218‐fold for 2.5(ZnMgNa)HAP and 130‐fold for 5Zn2.5(MgNa)HAP.

This exceptional activity is attributed to a synergistic mechanism: the release of bioactive Zn^2+^ ions combined with a significant pH increase (up to 10.0–10.5). Such a highly alkaline environment disrupts the metabolic activity and membrane integrity of *S. aureus*. Interestingly, the addition of Fe^3+^ to this system (2.5(FeZnMgNa)HAP) reduced its effectiveness (only a 20‐fold decrease at 20 mg/mL), likely due to a more moderate pH response (Figure 5e).

The composites exhibited varying results based on the metal in the ferrite phase (Figure 6f,g). Powder 2.5(MgNa)BCP‐25ZnFe remained almost inactive against *S. aureus*. This suggests that when Zn^2+^ is incorporated into the stable ferrite component rather than the phosphate phase, its bioavailability is diminished. Simultaneously, powder 2.5(ZnNa)BCP‐25MgFe showed a modest 4.6‐fold inhibition. These data indicate that a significant effect on *S. aureus* is achieved only when Zn^2+^ is part of the active, more soluble phosphate component, which facilitates the ionic release.

#### 3.5.2 Activity Against Pseudomonas aeruginosa

*P. aeruginosa* showed higher resistance to the tested materials compared to *S. aureus* (Figure 7). Fe^3+^‐containing samples (2.5FeHAP and 5FeHAP) and the co‐doped (Na^+^, Mg^2+^, Zn^2+^, Fe^3+^)‐hydroxyapatite (2.5(FeZnMgNa)HAP) showed negligible inhibition (less than 10‐fold) (Figure 7a,b,d). At the same time, concentration‐dependent effects were observed for the co‐doped (Na^+^, Mg^2+^, Zn^2+^)‐hydroxyapatites (Samples 2.5(ZnMgNa)HAP and 5Zn2.5(MgNa)HAP) in Figure 7c,d, respectively). At 20 mg/mL, growth was inhibited by 45.0 times for 2.5(ZnMgNa)HAP and 31.5 times for 5Zn2.5(MgNa)HAP. The some lower activity of sample 5Zn2.5(MgNa)HAP may be due to the presence of Zn^2+^ in ZnO form, which was established by XRD analysis.

The superior resistance of *P. aeruginosa* is likely due to its robust cell wall structure and highly efficient efflux pumps, which allow it to tolerate alkaline environments and metallic ions better than Gram‐positive *S. aureus*.

The distinct response of the two bacterial strains to the alkaline environment (pH) and the presence of Zn^2+^ ions is rooted in their fundamental cellular architectures. Gram‐positive *S. aureus* has a thick peptidoglycan layer, it lacks an outer protective membrane. This makes its cytoplasmic membrane highly vulnerable to the direct impact of OH^‐^ ions and Zn^2+^ cations. The high pH disrupts the proton motive force, leading to the dramatic 218‐fold growth inhibition observed in our study. Gram‐negative *P. aeruginosa* is notoriously resilient due to its outer membrane, which acts as a selective barrier. Furthermore, *P. aeruginosa* possesses sophisticated mechanisms for maintaining internal pH homeostasis under alkaline stress. This explains why, even at high sample concentrations, the maximum inhibition was limited to 45‐fold.

The antibacterial properties of these calcium phosphates are significantly enhanced by codoping with trace elements, particularly Zn^2+^. The mechanism of action is dual‐faceted: it involves the direct toxicity of released cations and, more importantly, the creation of an alkaline “shield” (pH approx. 10) during the partial dissolution of the bioactive phases. The influence of released ions on the bacterial cell membranes disrupts their normal physiological functions, compromising membrane integrity and ultimately leading to cell death. Furthermore, the extracellular lipid bilayer, which plays a crucial role in regulating cellular permeability, undergoes significant structural disorganization upon exposure to ion‐enriched antimicrobial agents. This disruption weakens the integrity of the cellular envelope and promotes the leakage of intracellular components [52, 53]. Electrostatic interactions and molecular forces facilitate the adhesion and anchoring of nanoparticles to the bacterial cell wall, representing a critical initial step in nanoparticle–cell interactions [54]. It has been shown that the degree of antibacterial activity, including the size of the inhibition zones produced by Fe‐HAp and Al‐HAp materials, is influenced by both the bacterial strain and the concentration of doping ions [53]. Thus, samples that demonstrate both a high zinc bioavailability and a strong pH‐increasing effect are the most promising candidates for suppressing postoperative bone infections.

### 3.6 Hemolisys Testing

**FIGURE 8:**
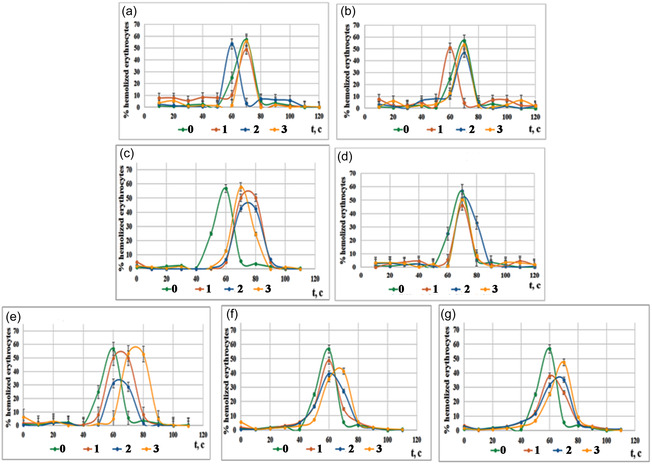
The dynamic of erythrocytes hemolysis for different amounts (1 mg/mL – (1), 2 mg/mL – (2) and 5 mg/mL – (3)) of prepared powders: 2.5FeHAP (a); 5FeHAP (b); 2.5(ZnMgNa)HAP (c); 5Zn2.5(MgNa)HAP (d); 2.5(FeZnMgNa)HAP (e); 2.5(MgNa)BCP‐25ZnFe (f); and 2.5(ZnNa)BCP‐25MgFe (g) (M ±m, *n* = 3).

To evaluate the biocompatibility and potential cytotoxicity of the synthesized materials, their impact on the resistance of erythrocytes to acid hemolysis was investigated. This method provides critical information regarding the presence of membrane‐damaging components and the overall interaction between the material's surface and cellular membranes. The resulting differential kinetic curves reflect the percentage of hemolyzed erythrocytes over time (Figure 8).

Modification of hydroxyapatite solely with Fe^3+^ ions led to a detectable decrease in erythrocyte resistance. Preincubation with 2 mg/mL of 2.5FeHAP or 1 mg/mL of 5FeHAP caused a leftward shift of the hemolysis curve, reducing the process duration to 90 and 80 s, respectively, compared to the control (Figure 8a,b).

This destabilizing effect may be attributed to the lower pH‐buffering capacity of these samples and the potential pro‐oxidant activity of Fe^3+^ ions at specific concentrations, which can induce lipid peroxidation in the erythrocyte membrane.

In contrast, samples co‐doped (Na^+^, Mg^2+^, Zn^2+^)‐containing hydroxyapatite (2.5(ZnMgNa)HAP and 5Zn2.5(MgNa)HAP) demonstrated a significant membrane‐stabilizing effect, shifting the hemolysis curves to the right (Figure 8c,d). The maximum hemolysis level dropped substantially: 31.32% for 2.5(ZnMgNa)HAP and 42.72% for 5Zn2.5(MgNa)HAP compared with the control (56.8%) (Figure 8c,d).

This stabilization correlates with the previously discussed a high alkaline reserve of these samples (pH approx. 10). In the context of an acid hemolysis test, the ability of these materials to increase pH and likely serves as a localized buffer, partially neutralizing the hemolytic agent (acid) and protecting the cells. Furthermore, Zn^2+^ and Mg^2+^ ions are known to interact with membrane phospholipids and proteins, increasing the mechanical rigidity and resistance of the lipid bilayer.

At the same time, the co‐doped (Fe^3+^, Na^+^, Mg^2+^, Zn^2+^)‐hydroxyapatite (2.5(FeZnMgNa)HAP) had a minor membrane stabilizing effect. In this case, the level of maximum hemolysis of erythrocytes decreased to 46.15% at its adding in the amount of 1 mg/mL compared with 56.88% for the control (Figure 8e).

The most pronounced protective results were observed for composites containing with 25 wt% of M^II^Fe_2_O_4_. The powder 2.5(MgNa)BCP‐25ZnFe (Zn‐rich) reduced maximum hemolysis by approximately 17% (at 2–5 mg/mL doses). At the same time, sample 2.5(ZnNa)BCP‐25MgFe (Mg^2+^‐rich) decreased hemolysis by 18.76% and 21.45% (at 1–2 mg/mL doses) (Figure 8f, g). The superior performance of these composites may be explained by the presence of the phase *β*‐Ca_3_(PO_4_)_2_ in the BCP‐based composites, which facilitates a controlled release of stabilizing cations and maintains a favorable alkaline microenvironment.

The results indicate that while pure Fe^3+^‐modification may induce slight membrane stress, the co‐doping with Mg^2+^ and Zn^2+^, especially in composite forms with ferrites, significantly enhances the cytoprotective properties of the materials. This protective effect may be a direct consequence of the dual‐action mechanism: neutralization of acidic stress via the increasing of pH and ionic stabilization of the erythrocyte membrane by divalent cations.

While our current study establishes the physicochemical and antibacterial foundation of the multidoped composites, future research should focus on long‐term in vivo studies to assess osseointegration and systemic biosafety beyond preliminary in vitro screens. Furthermore, the degradation kinetics of the BCP matrix must be tailored to match the rate of new bone ingrowth. From a mechanical standpoint, evaluating fatigue life under cyclic weight‐bearing loads is essential. Finally, optimization of the magnetic properties to ensure efficient hyperthermia within clinical safety limits will be a priority for subsequent developmental stages.

## 4 Conclusion

Fe^3+^, CO_3_^2‐^‐containing or complex co‐doped (Na^+^, Mg^2+^, Zn^2+^, CO_3_^2‐^)‐hydroxyapatites and composites based on modified calcium phosphates with 10 or 25 wt% of M^II^Fe_2_O_4_ (M^II^ – Mg^2+^, Zn^2+^) were successfully synthesized from aqueous solutions and heated to 600 °C. It was found that an increase of (M^2+^ + Fe^3+^) amount in initial mixtures caused the formation of composites modified with biphasic calcium phosphates with ferrite. Powders of co‐doped (Na^+^, Mg^2+^, Zn^2+^, CO_3_^2‐^)‐hydroxyapatites showed membrane stabilizing effect which was accompanied by a decrease of the maximum level of erythrocytes hemolysis. The greatest membrane stabilizing effect was found at adding of 2 mg/mL (Na^+^, Mg^2+^, Zn^2+^, CO_3_^2‐^)‐containing hydroxyapatites or 1 mg/mL of composite based on (Na^+^, Zn^2+^, CO_3_^2‐^)‐modified biphasic calcium phosphate with 25% MgFe_2_O_4_. These powders reduced erythrocytes hemolysis by 25.26% and 21.45% compared to the control, respectively. At the same time, the preincubation with Fe^3+^, CO_3_^2‐^‐containing hydroxyapatites decreased the erythrocytes resistance to hemolysis. Co‐doped (Na^+^, Mg^2+^, Zn^2+^, CO_3_^2‐^) hydroxyapatites had the highest antimicrobial activity against both *P. aeruginosa* and *S. aureus strains*.

The proposed one‐stage synthesis technique allows for the creation of multifunctional “smart” materials. By tuning the ratio of the ferrite phase and the degree of ionic substitution, it is possible to regulate the material's microhardness, magnetic response, and pH‐driven therapeutic activity. Thus, the developed composites are promising candidates for advanced bone tissue engineering. Specifically, their tailored magnetic properties open avenues for magnetic‐field‐guided drug delivery and hyperthermia‐induced cancer therapy. Furthermore, the combination of high Vickers hardness (up to 3.28 GPa) and antibacterial efficacy ensures that these materials can function as durable, infection‐resistant bone grafts. The biphasic composition also provides a tunable platform for bio‐resorbable scaffolds, where the dissolution rate can be adjusted to match the speed of new bone formation. Consequently, these materials can simultaneously combat infection, neutralize inflammation‐induced acidosis, and support mechanical stability during the regeneration process.

## Funding

This article was supported by the National Research Foundation of Ukraine (grant no. 2023.03/0109).

## Conflicts of Interest

The authors declare no conflicts of interest.

## Data Availability

The data that support the findings of this study are available from the corresponding author upon reasonable request.
